# Seroprevalence and molecular detection of brucellosis among Pakistani women with spontaneous abortion

**DOI:** 10.3389/fpubh.2024.1372327

**Published:** 2024-04-15

**Authors:** Mohammad Ejaz, Shahzad Ali, Muhammad Ali Syed, Falk Melzer, Rani Faryal, Maryam Dadar, Shahid Ahmed Abbasi, Hosny El-Adawy, Heinrich Neubauer

**Affiliations:** ^1^Department of Microbiology, Government Postgraduate College Mandian, Abbottabad, Pakistan; ^2^Department of Microbiology, Quaid-i-Azam University, Islamabad, Pakistan; ^3^Wildlife Epidemiology and Molecular Microbiology Laboratory (One Health Research Group), Discipline of Zoology, Department of Wildlife & Ecology, University of Veterinary and Animal Sciences, Lahore, Pakistan; ^4^Department of Microbiology, The University of Haripur, Haripur, Pakistan; ^5^Institute of Bacterial Infections and Zoonoses, Friedrich-Loeffler-Institut, Jena, Germany; ^6^Razi Vaccine and Serum Research Institute (RVSRI), Agricultural Research, Education and Organization (AREEO), Karaj, Iran; ^7^Department of Microbiology, Fauji Foundation Hospital, Rawalpindi, Pakistan; ^8^Faculty of Veterinary Medicine, Kafrelsheikh University, Kafr El-Sheikh, Egypt

**Keywords:** brucellosis, pregnancy, abortion, miscarriage, intrauterine fetal death

## Abstract

**Background:**

Human brucellosis is a neglected disease transmitted to humans from animals such as cattle, goats, dogs, and swine. The causative agents are bacteria of the genus *Brucella*, intracellular pathogens usually confined to the reproductive organs of their animal hosts causing sterility and abortions. The objective of the study was to determine the seroprevalence of brucellosis among women with spontaneous abortions (SAW) and compare this seroprevalence with that of healthy pregnant women (HPW).

**Methods:**

The case–control study was designed to determine the seroprevalence and molecular detection of brucellosis in women who suffered from spontaneous abortion and healthy pregnant women of the Haripur District of Pakistan. A total of 770 blood samples (*n* = 385 for each group) were collected from 9 public and 11 private hospitals in Haripur District from December 2021–March 2023. Data on demographic features, epidemiological variables, and risk factors were collected from each participant by structured questionnaires. Initial screening for brucellosis was performed by Rose Bengal Plate Test followed by qRT-PCR for molecular detection of the genus-specific *BCSP-31* gene of *Brucella*.

**Results:**

The study showed that anti-Brucella antibodies were more found in SAW 23.63% (91/385) than in HPW 1.29% (5/385). *Brucella* specific DNA was amplified in 89.01% (81/91) seropositive samples of SAW. Demographic features and risk factors such as age, urbanicity, socioeconomic status, education, occupation, and animal contact were found significantly associated with brucellosis (*p* ≤ 0.05). Consumption of unpasteurized raw milk (OR = 18.28, 95%CI: 8.16–40.94) was found highly concomitant with seroprevalence.

**Conclusion:**

This study reports the first evidence of involvement of brucellosis in spontaneous abortions in women of Pakistan. The study can be used to develop strategies for risk management during pregnancy, to raise awareness for brucellosis, and develop control programs.

## Introduction

Brucellosis is a zoonotic bacterial disease caused by the members of the genus *Brucella,* aerobic, non-motile, non-spore-forming, non-hemolytic, facultative intracellular and gram-negative coccobacilli (1). Being an intracellular pathogen, brucellae are usually confined to the reproductive organs of their animal hosts causing sterility and abortions. Brucellae can survive and multiply inside epithelial cells, dendritic cells, macrophages, and placental trophoblast (2). Four *Brucella* (*B.*) species are pathogenic to humans including *B. abortus*, *B. melitensis*, *B. suis*, and *B. canis* that can be transmitted from cattle, goats, swine, and dogs, respectively. *B. ovis*, *B. inopinata*, *B. neotomae* and *B. microti* are known to primarily infect animals but have not been documented to infect humans (3).

Brucellosis is usually transmitted to humans either by direct contact with animals or indirectly via the consumption of unpasteurized animal products like milk, cheese, butter and meat (4). Brucellae may be shedded in large numbers in milk, placental fluid, urine and other body fluids (5).

The disease burden is increasing in humans and bovines due to extensive urbanization, commercialization, trends in livestock farming, and unhygienic practices in animal husbandry and food handling (6). In 2021, approximately 227 million women worldwide become pregnant each year, with about one-third facing stillbirth, miscarriage or induced abortion for various reasons (7). The global burden of brucellosis is high and many studies regularly cited the incidence of 500,000 cases per year but this number seems to be underestimated due to lack of surveillance and underreporting in developing countries (8).

Brucellosis is endemic in many countries in Asia, the Middle East, Africa, Mediterranean Basin and Latin American countries with a higher incidence in central Asia and the Middle East (9, 10). In endemic countries such as India, Pakistan, Iran, Bangladesh, Italy, Spain, Greece, Southern France and Turkey, the prevalence of brucellosis have reached 10 cases per 100,000 of the population (11). The cumulative incidence of human brucellosis and seroprevalence in pregnancy cases per 100 delivered obstetrical discharges was reported to be 0.42–3.3% and 1.5–12.2% in endemic regions, respectively (12). The prevalence of brucellosis depends on the region and may range from 1.5 to 16.9% in pregnant women cohorts. The most common, dramatic, and unfavorable outcomes of brucellosis during pregnancies are miscarriages (1.2–29%), intrauterine fetal death (0–21%), or abortions (2.5–54%) (12).

Brucellosis is one of the neglected diseases in Pakistan because of a lack of effective control measures, awareness and surveillance data. A large number of the population of rural areas of Pakistan depend on livestock for their livelihood. Therefore, local populations are in direct contact with animals. Furthermore, the literacy rate in rural areas is very low and animal keepers or farmers have very little knowledge about the disease. Brucellosis is widespread in Pakistan, with numerous studies documenting the disease in various regions of the country (13–16). Seroprevalences of brucellosis were found to be 6.9% in high-risk occupations and 8.5% in pregnant women in district Lahore and Kasur of Pakistan (17). Similarly, a study conducted in a district Abbottabad reported overall seroprevalence of 13.6% in hospitalized patients having non-specific clinical signs and symptoms related to brucellosis (18). Moreover, Niaz et al. reported the seroprevalence of 27.4% brucellosis among suspected female patients in Malakand district of Khyber Pakhtunkhwa (KPK), Pakistan (19).

The prevalence of brucellosis among people living in close proximity to animals in rural Pakistan was found to be 16% (14). As the majority of the population of Haripur has either direct or indirect contact with livestock, a higher risk of acquiring brucellosis due to close contact with livestock may exist (20). Moreover, nearly 24% of pregnant women with a previous abortion history tested seropositive for brucellosis in the nearby city of Abbottabad (18). In Pakistan, about 2.25 million abortions occur *per annum* and the abortion rate is 50 per 1,000 women aged between 15 to 49 years (21).

Currently, there is limited data available on the association of human brucellosis in pregnant women with obstetric complications in Pakistan. The study was aimed to determine the prevalence of anti-*Brucella* antibodies among these women who have had spontaneous abortion in Haripur District and to identify risk factors for *Brucella* infection. This research will serve to fill in the gaps in our understanding of disease epidemiology in the region, provide important insight into the factors that contribute to the spread of disease and inform policy makers.

## Materials and methods

### Study area

**Figure 1:**
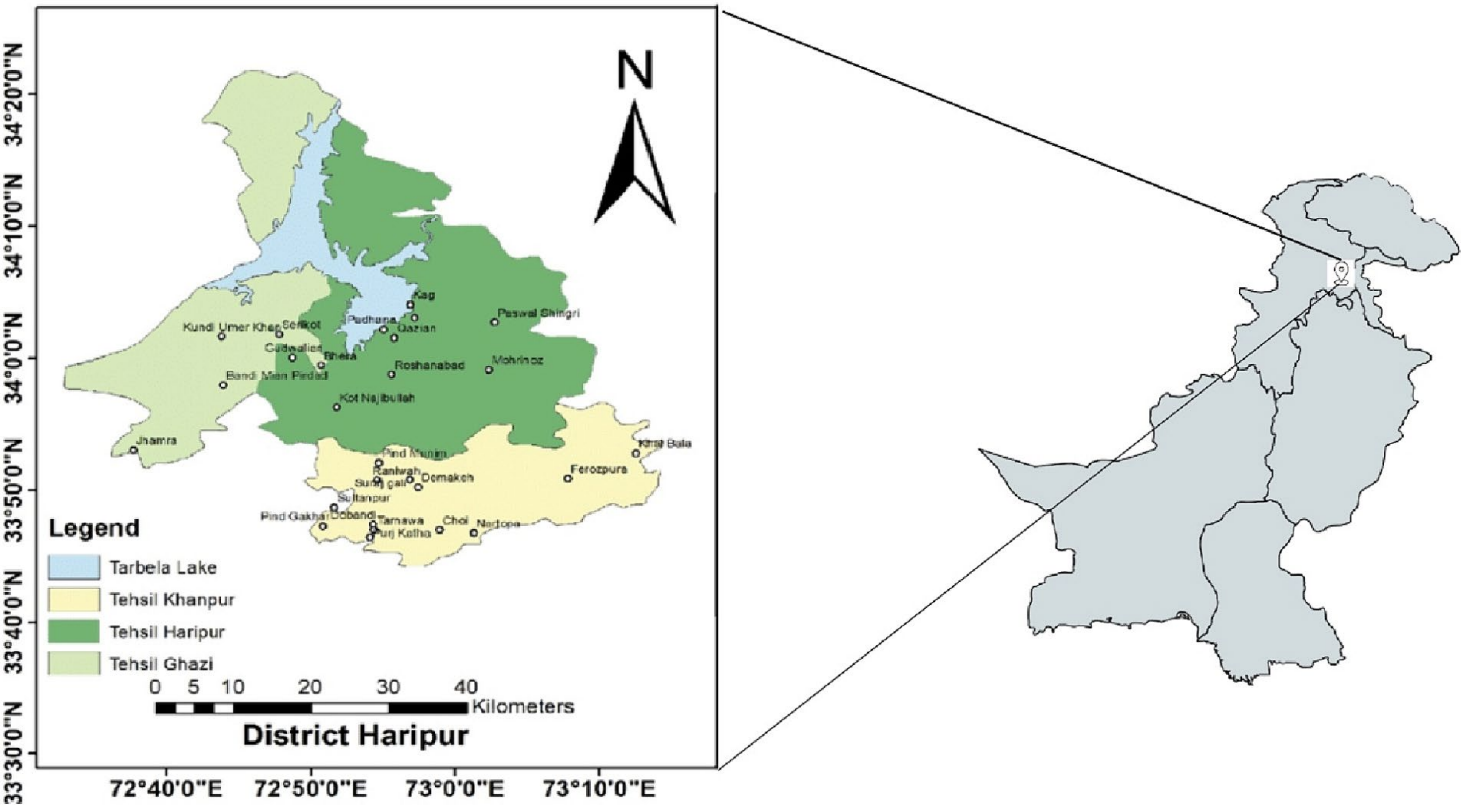
Sampling map of Haripur District of KPK, Pakistan.

The study was conducted at randomly chosen hospitals in Haripur, Pakistan, where gynecological care was provided. Haripur is located in the Hazara division and is one of the important cities of Khyber Pakhtunkhwa, Pakistan. Geographically, Haripur district borders Swabi to the west, Buner to the northwest, Abbottabad to the northeast, Punjab to the Southeast and lays approximately 33.9° north latitude and 72.9° east longitude with an altitude of 527 meters (Figure 1). Its total area is 796, 095 km^2^ or 666 square miles and is divided into three Tehsils including Khanpur, Haripur and Ghazi. According to the 2017 census, the total population of Haripur was 1,001,515 of which 868,415 (86.71%) were rural while 133,100 (13.29%) were urban population. The rural area of Haripur is fertile and suitable for agriculture and rearing of livestock. According to the livestock census 2006, district Haripur had more than 683,000 poultry and more than 459,524 ruminants including cattle (130,215), buffaloes (106,911), sheep (6,804) and goats (215,598) and 3,174 equidae, i.e., horses (894), mules (235) and asses (2,045) (22).

### Study design, participants and data collection

The case–control study was designed to determine the seroprevalence and molecular detection of brucellosis in women who suffered from spontaneous abortion (SAW, case group) and healthy pregnant (HPW, control group) women of the Haripur District of Pakistan. A total of 770 blood samples (385 from SAW and 385 from HPW) were collected during December 2021–March 2023 randomly from women visiting different public and private hospitals in Haripur District (Table 1). Women (Mean age: 28 ± 5.47 years) who were receiving medical care at healthcare facilities or visiting various laboratories for health complications or regular checkups provided the blood samples.

**Table 1:** Sampling distribution from two groups in different localities.

Location	Spontaneous abortion	Healthy pregnant
District Headquarter Hospital (DHQ) Haripur	99	110
Yahya Welfare Hospital Haripur	32	30
Tehsil Headquarter Hospital (THQ) Khanpur	69	35
THQ Hospital Ghazi	45	35
Basic Health Unit (BHU) Khoi Nara	19	20
BHU Halli	29	20
other hospitals and laboratories	92	135
Total (770)	385	385

The sample size (n) was determined by using the following formula:


$$n=\frac{Z^{2}.p.\left(1-p\right)}{C^{2}}$$

Where, Z is standard normal deviate (1.96) at 95% confidence interval, C is the confidence interval, i.e., 5% (0.05), and *p* is the proportion in the targeted population estimated to have brucellosis, i.e., 27.4% as reported by previous study in KPK province of Pakistan (19). The calculated sample size from the above formula was 306. However, we included 385 members in each group.

A self-administered structured questionnaire was used to collect patient details, including demographic features (age, urbanicity, socioeconomic status, education and occupation), contact with animals, contact with aborted materials of animals or humans, mode of animal contact, consumption of unpasteurized milk, number of pregnancies and spontaneous abortions and types of spontaneous abortions (miscarriage, early intrauterine fetal death and late intrauterine fetal death). The questionnaire was translated into local language (Urdu) to get the accurate information from the study participants.

### Inclusion and exclusion criteria

The case group comprised of females who underwent spontaneous abortion, while the control group consisted of pregnant females who were in good health and had no prior history of spontaneous abortions. The study excluded women who were not pregnant. The spontaneous abortion was categorized into miscarriages, early intrauterine fetal death and late intrauterine fetal death. Miscarriages are spontaneous abortions that occur before the 20th week of pregnancy, early intrauterine fetal death is the term for fetal deaths that occur between the 20^th^ and the 27^th^ week of pregnancy, and late intrauterine fetal death refers to fetal deaths that occur after the 28^th^ week of pregnancy.

### Blood collection

The brachial vein of the upper arm was located and disinfected by using a methylated spirit-soaked cotton swab. About 3 mL of blood was aseptically collected from the patient with a disposable sterile syringe. Blood was immediately transferred into EDTA and serum-separating gel vacutainers. The serum was separated by centrifuging the blood at 3,000 × g for 5 min (23). Each serum sample was transferred into a 1.5 mL sterile Eppendorf tube. The serum samples were stored at −4°C while blood samples were stored at 4°C till future use.

### Serology

Serum samples were subjected to Rose Bengal Plate Test (RBPT). RBPT is a rapid slide agglutination test used for the qualitative detection of anti-*Brucella* antibodies in human serum. A stained bacterial suspension (Veterinary Research Institute, Lahore, Pakistan) was used to detect anti-*Brucella* antibodies in the serum. An equal amount of undiluted serum and suspension (30 μL) was mixed on ceramic glossy white tile and was gently rotated for about 3–5 min. The serum was considered positive for brucellosis if agglutination was observed. The test was conducted on the day of sample collection to avoid any contamination.

### DNA extraction and quantification

DNA was extracted from seropositive blood samples using the Blood Genomic DNA Extraction Kit (Solarbio Science & Technology Co., Ltd. Beijing, China) according to the manufacturer’s protocols. The extracted DNA was quantified by measuring absorbance at 260 nm using a Nanodrop UV spectrophotometer (OPTIZEN NanoQ, K Lab Co., Ltd. Daejeon, South Korea). Quality of the preparation was analyzed (260/280 ratio) and testing was done with preparations containing ≥2 ng DNA /μl.

### Molecular detection through real-time polymerase chain reaction (RT-PCR)

The extracted DNA of seropositive samples was subjected to quantitative real-time PCR (qRT-PCR) for the detection of the *BCSP-31* gene. *BCSP-31* gene encodes a 31 kDa immunogenic membrane protein and is conserved among all *Brucella* species and biovars (24). The targeted gene and primers as well as the PCR thermal profile and conditions was previously demonstrated (18).

Briefly, a total of 25 μL volume of the reaction mixture was prepared for amplification using 12.5 μL of the optimized ready-to-use master mix [5X HOT FIREPol^®^ EvaGreen^®^ qPCR Mix Plus (ROX); Solis BioDyne] for real-time quantitative PCR assays containing HOT FIREPol^®^ DNA polymerase, MgCl_2_, dNTPs, EvaGreen^®^ dye and ROX dye, 0.8 μL forward primer (1 μM), 0.8 μL reverse primer (1 μM), 3 μL DNA template, and 7.9 μL of nuclease-free water.

The amplification of BSCP-31 and real-time fluorescence detection was performed on CFX96TM Real-Time System (C1000 Touch TM Thermal cycler Bio-Rad, United States). The first step was denaturation of the DNA template at 95°C for 10 min followed by the second step that had 44 cycles for 20 s at 95°C for denaturation, 50 s at 60°C for primer annealing, and 50 s at 72°C for DNA elongation.

### Statistical analysis

Data obtained through questionnaires were summarized and entered into Microsoft Excel 2013. A Chi-square test was used to analyze the association between test outcomes and risk associated factors using an online tool, Vassar Stat.^1^ Fisher exact test was used where the cross table (2 × 2 table) has five or less than five counts. Standard descriptive analysis was performed using Statistical Package for the Social Science (SPSS) version 22 to determine means and relative and absolute frequencies. Bivariate logistic regression analysis was performed to estimate the association between test outcome (seropositivity) and explanatory variables (information collected by questionnaire) using SPSS. The odds ratio and 95% confidence interval were also determined by bivariate logistic regression analysis. The data were considered statistically significant when the *p*-value was less than 0.05 (*p* < 0.05).

## Results

The study documented the various categories of spontaneous abortions experienced by each participant. Seroprevalence among women who suffered from spontaneous abortion was 23.63% (91/385) while in healthy pregnant women the seroprevalence was 1.31% (5/385). The seropositive samples were then subjected to genus specific gene (BCSP-31) qRT-PCR and the *Brucella* DNA was detected among 89.01% (81/91) in women suffered from spontaneous abortion and 80% (4/5) among healthy pregnant women. Tehsil-wise seroprevalence was also determined and found to be 25.3% (*n* = 33), 19.2% (*n* = 24), and 26.1% (*n* = 34) in saw from Khanpur, Haripur and Ghazi, respectively (Table 2).

**Table 2:** Tehsil-wise seroprevalence of brucellosis in the study participants.

Tehsil	Women with abortion history (*n* = 385)		Women with no abortion history (*n* = 385)	
	RBPT positive (%)	RBPT negative (%)	BBPT positive (%)	RBPT negative (%)
Khanpur	33 (25.3%)	97 (74.6%)	2 (1.5%)	128 (98.4%)
Haripur	24 (19.2%)	101 (80.8%)	1 (0.8%)	124 (99.2%)
Ghazi	34 (26.1%)	96 (73.8%)	2 (1.5%)	128 (98.4%)
Significance	Χ^2^ = 2.04, *p* value 0.36		Χ^2^ = 0.36, *p* value 0.85	

In this study, 24.8% (65/262) seroprevalence was found in women who had experienced miscarriages, 23% (15/65) in women with early intrauterine fetal death and 18.9% (11/58) with late intrauterine fetal death (Table 3). The present study revealed a positive correlation between the presence of anti-*Brucella* antibodies and an increased chance of spontaneous abortion during the initial stages of pregnancy.

**Table 3:** Demographic features and epidemiological variables of women who suffered from spontaneous abortion and had a positive Rose Bengal Plate test result.

Parameters	Variables	Total participants (*n* = 385)	Seropositive *n* = 91 (%)	Chi-Square (df)	*p*-value
Age^a^ (years)	17–24	118	21 (17.79)	34.77 (3)	<0.001
	25–32	182	32 (17.58)		
	33–40	63	33 (52.33)		
	>40	22	17 (22.72)		
Urbanicity	Urban	130	19 (14.61)	8.11 (1)	0.007
	Rural	255	72 (28.23)		
Socioeconomic status^b^	Low	208	60 (28.84)	8.04 (2)	0.048
	Middle	161	30 (18.63)		
	High	16	1 (6.25)		
Education	Illiterate	87	19 (21.83)	9.32 (3)	0.152
	Primary	109	37 (33.94)		
	Secondary	161	30 (18.63)		
	Graduate	28	5 (17.85)		
Occupation	Student	36	9 (25)	23.18 (4)	0.742
	Housewife	182	31 (17.03)		
	Farmer	63	21 (33.33)		
	Animal keeper	71	28 (39.43)		
	Others	33	2 (6.06)		
Animal contact	Yes	238	63 (26.47)	6.34 (1)	0.011
	No	147	22 (14.96)		
Mode of animal contact	Direct	80	28 (35)	14.48 (3)	0.187
	Indirect	97	29 (29.89)		
	Occasional	61	12 (19.67)		
	No	147	22 (14.96)		
Consumption of unpasteurized milk	Yes	112	61 (54.46)	80.77 (1)	<0.001
	No	273	30 (10.98)		
Contact with the animals during parturition^c^	Yes	134	23 (17.16)	4.24 (1)	<0.001
	No	251	68 (27.09)		
Contact with the person who had abortion^c^	Yes	56	5 (8.92)	6.93 (1)	0.003
	No	329	86 (26.13)		
Number of pregnancies	1	65	13 (20)	7.75 (3)	0.116
	2	115	19 (16.52)		
	3	125	39 (23.2)		
	>3	80	20 (25)		
Number of abortions	1	158	32 (20.2)	3.03 (2)	0.898
	2	137	32 (23.35)		
	>2	90	27 (30)		
Types of spontaneous abortions	Miscarriage	262	65 (24.8)	0.91 (2)	0.897
	Early Intrauterine fetal death^e^	65	15 (23)		
	Late intrauterine fetal death^f^	58	11 (18.9)		

^a^mean age = 28 ± 5.47 years, ^b^socioeconomic status based on monthly income (Low = <20,000 per month (PM), Middle= >30,000–200,000 PM, High= >200,000 PM), ^c^any contact with aborted materials or fluids of animals, ^d^before 20^th^ week of pregnancy, ^e^fetal deaths between 20–27^th^ weeks of pregnancy, f: fetal deaths after 28^th^ week of pregnancy.

Various risk associated factors including demographic features and epidemiological variables were identified (Table 3). A higher seroprevalence was identified in patients of the age group 33–40 years (52.33%) and > 40 years (22.72%). Education, socioeconomic status and occupation were statistically significant risk factors for seroprevalence (*p* ≤ 0.05). Seroprevalence was found to be higher in individuals who had not received any formal education (21.8%) or had only completed primary education (33.9%). Women from lower socioeconomic backgrounds and from middle-income families had seroprevalence of 28.8% and 18.3% and proved to be more vulnerable to disease than women with a high socioeconomic status. Occupations such as animal keepers (39.4%) and farmers (33.3%) were identified as significant risk factors (*p* = 0.0001). Consumption of unpasteurized milk and direct interaction with animals had a positive association with a positive RBPT result. This study revealed a higher seroprevalence in women with a higher number of pregnancies and a history of abortion, in comparison to women with fewer pregnancies and no history of abortion (Table 3).

The bivariate logistic regression analysis (Table 4) was performed to determine the relationship between various risk factors and seroprevalence in women who had animal contact or consumed unpasteurized milk. The highest seroprevalence was observed in patients who consumed unpasteurized milk (odds ratio = 17.90, 95%CI = 7.95–40.31). Similarly, the seroprevalence was higher in age groups 33–40 years (52.33%) and > 40 years (22.72%), and an odds ratio of 2.02 was observed in these age groups (95%CI = 1.35–3.01).

**Table 4:** Analysis of various features and their association with positive Rose Bengal Plate Test results using bivariate logistic regression analysis and odds ratio at 95% confidence interval (CI) with lower and upper limit and two-sided *p* value (0.05).

Variables	Odds ratio (95%CI)	95% CI	*p*-value
Age	2.02	1.35–3.01	<0.001
Urbanicity	2.68	1.30–5.53	0.007
Socioeconomic status	0.51	0.27–0.99	0.481
Education	1.41	0.88–2.25	0.152
Occupation	0.95	0.70–1.28	0.742
Animal Contact	1.13	0.30–4.2	0.897
Consumption of unpasteurized milk	17.90	7.95–40.31	<0.001
Contact with aborted materials of animals	0.12	0.12–0.46	<0.001
Contact with aborted materials of humans	0.18	0.06–0.56	0.003
Total number of pregnancies	0.69	0.44–1.09	0.116
Number of abortions	1.03	0.60–1.78	0.898

## Discussion

Brucellosis is an important zoonotic disease that is distributed all over the world. It is reported from more than 170 countries with over six million patients (25). Globally, about 500,000 new cases are reported per year; however, this number may be underestimated and the true incidence of cases might range from 5 to 12.5 million cases every year (26, 27). Brucellosis negatively affects human and animal health contributing to the disease burden in endemic regions and posing threat to animal production (28). Brucellosis is also endemic in Pakistan and many studies have reported the presence of brucellosis in both animals and humans. There are, however, few studies available that deal with the prevalence of brucellosis in women who had spontaneous abortions (13). The current study was done to investigate role of brucellosis in spontaneously aborting women in district Haripur.

Using RBPT this study found a seroprevalence of 23.63% (*n* = 91 out of 385) in women who had spontaneous abortions and 1.31% seroprevalence (*n* = 5 out of 385) in women with no history of spontaneous abortion. The present study shows an alarmingly high seroprevalence among women with spontaneous abortion history which is in concordance with a study done by Hassan and coworkers reporting a seroprevalence of 23.8% in patients with abortion history and clinical signs and symptoms of brucellosis (18). The higher prevalence of disease in women with spontaneous abortion history may be attributed to various risk factors including close contact with animals and lack of proper health care during pregnancy. Pregnant women are more prone to infections due to hormonal imbalance and changes in the immune system (29). Another study from Pakistan reported a prevalence of 24% (n = 6) in seropositive pregnant women with an abortion history. In these seropositive women, the study also reported that 12% of cases had intrauterine fetal death as a cause of spontaneous abortion (30). Similar results were also reported in a study from Rwanda, which recorded a seroprevalence of 25% in women who had obstetric complications such as abortion or stillbirth (31). Studies from Saudi Arabia also reported 27.3 and 46% prevalence of brucellosis in pregnant women who had obstetric problems such as spontaneous abortion (32, 33). The results of the current study cannot be compared to results of studies from neighboring India where only 4.2% and 9% prevalence of brucellosis was observed in aborted women with limited animal contact (34, 35).

Real-time PCR was used to molecularly detect *Brucella* in seropositive samples. Approximately 89% (81/91) of the samples tested positive for *Brucella*, demonstrating reduced sensitivity in comparison to the serological assay or false-positive results of serology. Considering the well-documented issue of significant serological cross-reactions with other bacteria, it is highly probable that many of these samples were false positives (36–38). The literature presents conflicting findings on the sensitivity and specificity of PCR analysis in detecting infected cases when compared to serological testing. While some research suggests that PCR is more sensitive and specific than serological tests for diagnosing brucellosis, other studies have shown that serological testing has a higher sensitivity (39–42). In our investigation, the serological testing showed greater sensitivity as indicated by a higher positive rate for the RBPT compared to PCR analysis. Utilizing both molecular and serological approaches enhances sensitivity for detecting positive cases, so it is recommended to employ both techniques.

Various demographic, epidemiological variables and risk factors were analyzed to predict the relationship between these factors and obstetric outcome in pregnant women with positive RBPT results. Indeed, higher seroprevalences were found in middle aged women. The higher prevalence registered in the middle age group may be caused by prolonged close contact with livestock and weakening of their immune system that could result in obstetric complications. The results of the present study vary significantly from those of a previous study involving pregnant Pakistani women. That study reported a higher seroprevalence in lower age groups 18–25 (44%) years and 26–33 years (28%) compared to that of the higher age groups (30). Similarly, a higher prevalence of brucellosis was found in the age group of 15–45 years in a study conducted in Kenya (43). Researchers in the nearby Abbottabad district of Pakistan also discovered brucellosis in older adults, although it was more common in the middle-aged population (18). The reason for such finding can be attributed to the fact that the members in this middle-aged group were primarily involved in activities such as animal’s milking, working in animal husbandries who had frequent and direct contact with animals. Nevertheless, individuals of all age groups were found to have seropositive instances of brucellosis.

In the present study, patients from rural areas were at higher risk due to direct contact with animals than women from urban regions. These findings are in accordance to results of several previous studies (18, 30, 44). This work reports two times higher seroprevalence in the study groups of rural regions than in the study group of the urban region of district Haripur. A similar study in Uganda had comparable results reporting approximately three-time higher prevalence in rural (21.4%) compared to urban regions (7.9%) (45). The higher prevalence in people of rural areas is caused by routine practices including herding of livestock, close contact with animals, birthing of calves, and consumption of raw animal products putting them at high risk of getting infected by *Brucella* (14).

Knowledge and awareness about brucellosis are important for controlling the spread of the disease and education of individuals is an important factor in limiting its incidence. Better education *per se* provides better knowledge and attitudes toward hygiene of individuals to prevent them from acquiring diseases like brucellosis. In the present study, the seroprevalence of brucellosis was higher in women who had no (21.83%) or only primary (33.94%) education in contrast to those with secondary (18.63%) and higher education (17.85%). This study is comparatively consistent with a previous study from Uganda that reports a prevalence of 5.7% in persons who had tertiary level of education (46).

The present study also investigated women having spontaneous abortion in respect to their occupations and found a higher prevalence in women who were animal keepers (43.66%) followed by farmers (38.09%). The study is consistent with previously reported studies from countries that also detect higher prevalence in persons with occupations like animal keepers, veterinarians, dung makers, and milkers (17). Smita et al. also reported 22.08% (36/163) prevalence of brucellosis in shepherds rearing livestock in India (47). A cross-sectional study from Kenya by Makala et al. also reported a prevalence of 10.4% (*n* = 31) among individuals who were agro-pastoralist (48).

Several studies have reported animals as an important risk factor for human brucellosis and the presence of *Brucella* in infected animals contributes significantly to causing transmission (13). Brucellae can be transmitted to humans via contact with infected animals, consumption of unpasteurized milk, and contact with aborted fetuses or products thereof of infected animals or humans. Regular contact with humans with animals is considered the primary source of infection with *Brucella,* while consumption of unpasteurized dairy products is the secondary source of *Brucella* infections in various endemic countries (49, 50). The current study reported that contact with animals contributes significantly to causing human brucellosis in susceptible populations (χ^2^ = 14.48, *p* = 0.0118). The participants who had direct contact with animals were more susceptible to acquiring brucellosis compared to individuals who had no or occasional contact with animals. Current results are contradictory to the previously reported prevalence of 36% (*n* = 9) in pregnant women who had animal contact while 64% (*n* = 16) in women with no animal contact (30). It is possible that the lower prevalence in the previously reported studies was influenced by regional factors or a smaller study population. In contrast, the higher prevalence in our present study can be attributed to a larger sample size and the endemic nature of animal brucellosis in the region. However, Copper et al. reported an odds ratio of 2.82 (95% CI = 1.03–3.04) among seropositive individuals who had direct contact with animals (51). The present study reports a prevalence of 54.46% (*n* = 61) with an odds ratio of 18.28 (95% CI = 8.168–40.941) in women who consume unpasteurized milk. Previous studies from countries targeting various populations including pregnant women also reported similar results of acquiring brucellosis via consumption of unpasteurized milk (14, 17, 18, 30). This study finds high correlation of brucellosis occurrence in individuals who had direct contact with animals and consume unpasteurized milk which was not unexpected.

Close contact with the aborted material or genital discharge of animals and humans was also recorded for participants. A seroprevalence of 17.16% (*n* = 23) was observed in women who had close contact with aborted animals during parturition. Similarly, 8.92% (*n* = 5) prevalence of brucellosis was observed in women who had contact with aborted material and fluids of parturient humans having spontaneous abortion. Indeed, several studies reported a higher prevalence of disease in individuals who had contact with animal or human abortions (52–54). Makala et al. also reported a higher odds ratio of 3.1 (95%CI = 1.18–8.37) in individuals who had assisted animals during parturition and had direct contact with animal placentas (48). Therefore, it is important for midwives to prioritize environmental hygiene, use personal protective equipment, and practice proper hand hygiene to prevent any infections.

The number of pregnancies and number of abortions is also recorded in women who suffered from spontaneous abortions. A higher prevalence of brucellosis in women was registered who had more pregnancies and a higher rate of abortions. However, no significant relationship between the number of pregnancies and abortion with seroprevalence was found. Similarly, Kurdoglu et al. reported corresponding results in their study in Turkey, where a higher prevalence of brucellosis was documented in women who had ≥3 pregnancies (48.15%) (55). Several other studies also reported a missing relationship between the number of abortions and the prevalence of brucellosis. The reason for inconsistent data may be due to several other factors associated with brucellosis or spontaneous abortions of other causes (33, 56).

The present study also recorded information on the types of spontaneous abortion and determined the seroprevalence of brucellosis for the different groups. The current study shows seroprevalence of 24.8% in miscarriages, 23% in early intrauterine fetal death, 18.9% in late intrauterine fetal deaths as a cause of spontaneous abortion. The present study reported a higher seroprevalence of brucellosis in miscarriages than previously reported for different countries (57–59). It was observed in the current study that the chances of abortion were higher if women suffered from brucellosis during the early stage of their pregnancies. The rate of miscarriages due to brucellosis ranges between 1.2% (*n* = 3/242) (59), 9.1% (*n* = 1/9) (58), 14.0% (*n* = 12/86) (60), 17.9% (*n* = 7/39) (57) and 28.6% (*n* = 2/7) (12) in different studies. Previously reported studies in different countries reported that intrauterine fetal death due to brucellosis rates between 0% and 20.6% (57, 61). Our findings fit very well in this scenario.

Due to the complex nature of manifestations associated with brucellosis, many signs and symptoms are non-specific. Moreover, physicians often fail to make a timely diagnosis of the disease and thus misdiagnosis can occur which can lead to severe complications. Therefore, it is necessary to make an accurate and precise diagnosis of the disease using serological and molecular techniques. RT-PCR is a rapid, highly sensitive, and specific method for molecular diagnosis as compared to other conventional methods avoiding risk for operators during cultivation. The combination of RBPT with RT-PCR helps to provide accurate and precise diagnosis of brucellosis. However, more research has to be done to compare RBPT and cultivation vs. RBPT and RT-PCR to decide on the best diagnostic strategy.

The present study has certain limitations including that it primarily relies on RBPT as sole serological assays. Although, RBPT is valuable and highly sensitive assay, it is imperative to further add confirmatory assays such as Complement Fixation Test (CFT), Enzyme-linked Immunosorbent Assay (ELISA), or *Brucella* Capt test to improves the reliability and accuracy of the results. Additionally, the present study’s utilization of serological assay and RT-PCR analysis is restricted to detect only *Brucella* genus and no attempt were made to isolate and identify the specific *Brucella* species responsible for causing spontaneous abortions in pregnant women.

Consequently, it is important to conduct further research to elucidate the prevalent species of *Brucella* in the population. Additionally, the molecular epidemiology of circulating *Brucella* species within Pakistani population remains unexplored. Therefore, comprehensive studies are imperative to unravel the molecular epidemiology, including genotyping characterization and phylogenetic analysis to gain insights into the genetic diversity and transmission dynamics of *Brucella* strains in the high-risk population.

## Conclusion

The study determined the seroprevalence of brucellosis among healthy pregnant women and women who suffered from spontaneous abortions in the Haripur District of Pakistan. The study showed that brucellosis is more prevalent in women who had a history of spontaneous abortion compared to healthy pregnant women. Moreover, risk factors like animal contact and consumption of unpasteurized milk may cause brucellosis infection in pregnant women. High-risk individuals like pregnant women must be made aware of the zoonotic aspect of the disease and need to be educated about precautionary measures. Tests to detect anti-*Brucella* antibodies in pregnant women of endemic areas need to be done on routine basis to avoid any obstetric complications. These data can be used for further research on correlations between brucellosis and human abortion. The results of the present study can also be used to develop strategies for controlling human brucellosis, raise awareness about the obstetric risks in pregnant women, and develop eradication programs in the study region.

## Data availability statement

The raw data supporting the conclusions of this article will be made available by the authors, without undue reservation.

## Ethics statement

The studies involving humans were approved by Ethical review committee of University of Haripur, Pakistan. The studies were conducted in accordance with the local legislation and institutional requirements. The participants provided their written informed consent to participate in this study.

## Author contributions

ME: Writing – original draft, Writing – review & editing, Data curation, Investigation, Validation. SA: Conceptualization, Formal analysis, Methodology, Writing – original draft, Writing – review & editing. MS: Conceptualization, Methodology, Writing – review & editing. FM: Funding acquisition, Investigation, Methodology, Resources, Writing – review & editing. RF: Conceptualization, Methodology, Supervision, Writing – review & editing. MD: Formal analysis, Investigation, Methodology, Software, Writing – review & editing. SAA: Investigation, Methodology, Validation, Writing – review & editing. HE-A: Conceptualization, Funding acquisition, Methodology, Resources, Software, Writing – review & editing. HN: Conceptualization, Formal analysis, Funding acquisition, Methodology, Resources, Writing – review & editing.

## Footnotes

^1^ http://vassarstats.net/
